# Numerical simulation study on inferring the location of subsurface karst water channels using shallow geothermal field data

**DOI:** 10.1371/journal.pone.0336852

**Published:** 2025-11-26

**Authors:** Qing Zhang, Renzhe Zeng, Zongfang Chen, Yunfeng Li, Weiya Ge, Yuanzhi Lu, Junhao Qiu

**Affiliations:** 1 Geological Survey Center, China Geological Survey, Nanjing, China,; 2 SHAANXI LIZHENG Investigation and Design Co., LTD, Xi'an, China,; 3 College of Construction Engineering, Jilin University, Changchun, China; Khalifa University, UNITED ARAB EMIRATES

## Abstract

Karst structures significantly impact the environment and engineering projects. The presence of water-bearing karst structures alters the shallow stratigraphic temperature field. The shallow temperature measurement method offers a simple and efficient approach to obtain shallow ground temperature data, enabling the inference of karst structure distribution through temperature anomalies. In this study, the feasibility of using shallow thermometry to detect karst pipeline structures was investigated via numerical simulation at the Hongsheng Coal Coking Plant and its surrounding sites in Panzhou City, Guizhou Province, China. The results indicate that variations in the burial depth of karst structures markedly influence shallow stratum temperatures. For a single karst conduit with an equivalent diameter of 0.5 m and water temperature of 12 °C, the detectable depth limit is approximately 66 m. Although an increase in the effective flow cross-sectional area affects shallow stratigraphic temperatures, changes in equivalent diameter under the site-specific conditions alter the temperature at 2 m depth by less than 0.02 °C, making it difficult to identify the effective flow cross-sectional area using shallow thermometry. Variations in fluid temperature within a certain range (12–18 °C) also affect shallow ground temperatures, with the influence of lower-temperature fluids being more pronounced. This study provides a rapid, cost-effective, and relatively accurate method for investigating subsurface karst structures, offering important implications for related engineering applications.

## 1. Introduction

Approximately 15% of the Earth’s ice-free land surface consists of karst regions [1,2]. In soluble formations, the dissolution and erosion of certain minerals by groundwater enlarge pores within the rock mass, enhancing seepage capacity and potentially forming interconnected karst channels with dynamic water flow [3,4]. Under specific geological conditions, such water-bearing karst structures may trigger geohazards, including landslides, dam leakage, piping erosion, and leachate contamination from landfills. Therefore, accurate detection of these karst systems is critical for geohazard prevention and mitigation.

The combination of geophysical exploration and drilling is a widely adopted approach for investigating karst structures. Common geophysical techniques include high-density electrical resistivity tomography (ERT), shallow high-density seismic reflection, cross-hole tomography (CT), transient electromagnetic method (TEM), Rayleigh surface wave detection, and borehole televiewer logging [5–8]. Electrical and seismic methods effectively detect water-bearing strata by leveraging differences in electrical properties and elastic wave velocities between soil and groundwater. However, when applied to “vein-type” water-bearing karst structures, these methods face limitations due to the similar resistivity and seismic wave velocities between the flowing channels and the surrounding water-seeped zones, making it difficult to accurately delineate the pathways of groundwater flow. In addition to geophysical techniques, direct detection methods such as tracer tests can provide explicit groundwater flow information [9,10]. While these methods yield valuable hydrogeological data, their effectiveness relies on the accurate prediction of conduit locations and flow paths, which is inherently dependent on the selection of sampling points. Moreover, tracer tests are time-consuming, costly, and carry the risk of water contamination, limiting their widespread applicability.

Given the constraints inherent in conventional methodologies, it becomes imperative to develop an innovative investigation technique that enables efficient, cost-effective, and eco-friendly detection of aqueous karst structures exhibiting “flow vein” configurations. The shallow geothermal prospecting method represents a geophysical surveying approach specifically developed for characterizing subsurface thermal distribution patterns in shallow geological formations. Through systematic acquisition and analysis of surface and near-surface thermal data, this methodology facilitates the interpretation of thermal anomalies in shallow subsurface environments. Its application spectrum encompasses multiple domains such as groundwater resource assessment, geothermal energy exploitation, and geohazard evaluation [11–14]. This method is relatively simple, cost-effective, and capable of providing accurate temperature data for shallow strata, offering critical support for related research and engineering decision-making. The thermophysical properties of rock masses depend on lithology, temperature, and structural characteristics. In certain cases, rock mass structure governs its thermophysical behavior. Due to structural heterogeneity, rock masses are treated as continuous media, with equivalent thermophysical properties used to analyze their temperature fields [15]. Studies indicate that heat transfer in water-bearing karst structures primarily occurs through conduction and convection [16–18], and their presence significantly alters the surrounding strata’s thermal distribution [19]. This makes shallow geothermal prospecting a viable method for detecting such structures. By utilizing shallow subsurface temperature measurements, this approach provides an efficient, economical, simple, and rapid means of investigating water-bearing karst formations.

Shallow geothermal prospecting is a highly efficient and practical geophysical exploration method. Research has shown that in the variable-temperature layer, the influence of seasonal changes, vegetation, and solar irradiation on subsurface temperature diminishes with increasing depth. Crucially, temperature variations at 1-meter depth exhibit negligible diurnal solar-induced fluctuations [20]. Despite potential disturbances from precipitation and vegetation, thermal signals at this depth can still reflect heat sources as deep as 400 meters [21]. Initially employed by Japanese scholars in the 1940s for thermal spring exploration [22], this method underwent significant theoretical refinement in the 1960s [20,23]. Subsequent work elucidated the effects of environmental factors (e.g., diurnal/annual cycles, groundwater flow) on shallow thermal regimes [24]. Cartwright established a correlation between thermal patterns and groundwater movement, enabling the delineation of subsurface flow pathways [25]. Takeuchi Atsuo later adapted the technique for groundwater detection in landslide-prone areas, and Cheremensky extended its applications to geotechnical engineering [23]. Therefore, these case studies demonstrate the feasibility of utilizing shallow geothermal measurements to delineate the scale and spatial orientation of subsurface karst structures, while effectively circumventing the high costs associated with conventional geophysical exploration methods.

Numerical simulations are employed to analyze the influence of water-bearing karst structures on the distribution of shallow geothermal fields and to assess the feasibility of using shallow geothermal measurements for karst structure investigations. Analytical solutions are only applicable to simple geological conditions. For complex scenarios, numerical methods such as the finite element method (FEM) are typically used to address geological problems with intricate boundaries. With advancements in computer technology, numerical simulation software such as COMSOL Multiphysics, Midas, PFC, FLAC^3D^, Fluent, and Visual Modflow is frequently employed for addressing karst groundwater issues. Qiao et al. used numerical simulations to quantitatively characterize the seepage characteristics of karst geothermal reservoirs, providing an important basis for geothermal development in karst areas [26]. Employing COMSOL Multiphysics, He et al. investigated water inrush issues in karst tunnels, including their characteristics, mechanisms, and treatment measures, thereby offering critical insights for mitigating such geological hazards [27]. Song et al. combined field hydrogeological data with MODFLOW simulations to evaluate how tunnel construction affects karst aquifers [28]. Li et al. employed the finite element method to couple seepage flow in the karst matrix with free flow in the conduits. This work established a numerical model for water exchange at the microscale between the matrix and adjacent conduits, offering valuable insights for optimizing karst system prediction and management [29]. Therefore, the use of numerical simulation to analyze the distribution of temperature fields induced by heat transfer between deep subsurface fluids and strata has become widespread, as this approach can intuitively reveal anomalous temperature distributions underground.

This study focuses on a karst region in Panzhou City, Guizhou Province, China, investigating the application of 2-meter shallow geothermal measurement technology for detecting and characterizing shallow water-bearing karst structures. Through numerical simulations of thermal field distribution characteristics in these karst formations, we estimate key parameters of the karst structures based on the established numerical models. The geothermal data are quantitatively analyzed to determine their scale, depth, and flow pathways. This innovative application of shallow temperature measurement represents an indirect geological prospecting approach, providing a rapid and cost-effective investigation method for karst water resource development and geohazard prevention.

## 2. Fundamental theories

### 2.1 Fundamentals of shallow stratum thermometry

This study employs the 2-meter thermal probe method (2m-TPM) developed by Zhang et al [23]. This methodology, based on the thermal needle probe principle derived from Fourier’s law of heat conduction, represents a transient measurement approach. Its core theoretical model involves an analytical solution of the temperature field response generated by an infinite linear heat source within a homogeneous, semi-infinite medium under controlled heating conditions. Consider an infinitely long, straight heating wire with radius r_0_ embedded in an infinite, homogeneous, and isotropic medium, establishing a one-dimensional cylindrical heat transfer model. The medium initially maintains a uniform temperature T_0_, with thermal conductivity λ, thermal diffusivity α, density ρ, and specific heat capacity *c*. When a heating wire of length L is vertically inserted into the medium and begins to generate heat at a constant rate *q* (W·m ⁻ ¹) uniformly from time t = 0, transient heat conduction occurs radially outward from the wire axis. Defining the excess temperature as θ = T - T_0_, the temperature field within the medium satisfies the following governing differential equation and initial conditions:


$$\{\begin{array}{c} {}*20c\frac{\partial \theta }{\partial t}=a(\frac{\partial^{2}\theta }{\partial r^{2}}+\frac{\text{1}}{r}\frac{\partial \theta }{\partial t})\;\;\;\;\;t>0,0<r<\infty \\ \theta (r,t){|}_{r=0}=0\\ \begin{array}{c} {}*20c\theta (r,t)=0\;\;\;\;\;\;\;\;\;\;\;\;\;\;t>0,r\to \infty \\ q=-2\pi \lambda r_{0}{\left(\frac{\partial \theta }{\partial r}\right)}_{r=r_{0}} \end{array} \end{array}$$ (1)

The temperature field in the measured medium exhibits cylindrical isothermal surfaces centered on the heating wire axis. The temperature rise in the medium progressively decreases with increasing radial distance, eventually stabilizing at a certain radius *r* from the heating wire. Through temporal discretization of the interval [0, t] into differential elements, the thermal wire is modeled as an ordered continuum of instantaneous point sources, with each source term characterized by the transient heat release rate q(t')dt'. For an elemental time duration dt' at a given instant t', the induced temperature variation dT in the test medium over the time span t-t' can be mathematically represented as:


$$dT=\frac{\frac{q(t^{\prime })}{\rho c}dt^{\prime }}{\text{4}\pi a(t-t^{\prime })}exp\left[-\frac{r^{\text{2}}}{\text{4}a(t-t^{\prime })}\right]$$ (2)

At time t = t', the temperature distribution at radial position *r* can be calculated by the following equation:


$$\theta \left(r,t^{\prime }\right)=\frac{q/\rho c}{\text{4}\pi a(t-t^{\prime })}exp[-\frac{r^{\text{2}}}{\text{4}a(t-t^{\prime })}\backslash ]$$ (3)

### 2.2 Thermodynamic Coupling in Karst Aquifers

#### 2.2.1 Thermal transport in karst conduit flows.

The presence of water-bearing karst structures at certain depths can substantially modify the thermal distribution characteristics in shallow strata, primarily through the cooling effects induced by continuous groundwater flow within karst channels. The heat exchange process is predominantly controlled by multiple factors, including the mean fluid temperature within karst channels, the ambient rock temperature, the thermal resistance of the rock matrix, and the convective thermal resistance between groundwater and the host rock. The conduit systems in karst formations typically evolve from joints and fractures through prolonged dissolution processes. In this numerical investigation, we focus specifically on the thermal impact of individual karst channels, while deliberately neglecting the influence of fracture network flow to simplify the modeling complexity. Based on the flow pathways and patterns in karst conduit systems, the hydrodynamic behavior can be conceptually simplified as pipe flow for analytical purposes. This simplification allows the derivation of a single-conduit flow equation using the fundamental equations of uniform flow, as follows [30,31]:


$$u=K_{C}\cdot J_{C}$$ (4)

where *K*_*c*_ represents the conduit permeability coefficient, m/d; and *J*_*c*_ denotes the hydraulic gradient within the conduit.

In addition, the momentum and continuity equations for flow in a pipe are given by [32]:


$$\rho \frac{\partial u}{\partial t}+\rho u\cdot \nabla u=-\nabla \text{p}-f_{D}\frac{\rho }{\text{2}d_{h}}u\left|u\right|+F$$ (5)

And


$$\frac{\partial A\rho }{\partial t}+\nabla \cdot \left(A\rho u\right)=\text{0}\;$$ (6)

The second term on the right-hand side in Equation (5) represents the pressure drop due to viscous shear. Here, u is the cross-section averaged velocity (SI unit: m/s), ρ the density (SI unit: kg/m^3^), p pressure (SI unit: Pa), f_D_ (dimensionless) the Darcy friction factor and F is a volume force term (SI unit: N/m^3^).

Furthermore, dh is the mean hydraulic diameter (SI unit: m), given by:


$$d_{h}=\frac{\text{4}A}{Z}$$ (7)

where *A* is the pipe cross section area (SI unit: m^2^) available for flow, and *Z* is the wetted perimeter (SI unit: m).

The Darcy friction factor in Equation (5) accounts for the continuous pressure drop along a pipe segment due to viscous shear, and is expressed as a function of the Reynolds number (*R*_*e*_) and the surface roughness *e* divided by the hydraulic diameter (*e/d*_*h*_).


$$f_{D}=f_{D}\left(Re,\frac{e}{d_{h}}\right)\;$$ (8)

The Reynolds number *Re* is calculated using the formula *Re = ρudh/μ*, where *μ* represents the dynamic viscosity coefficient of the fluid.

Assuming the flow of groundwater in karst channels is incompressible, the energy equation governing the flow satisfies the following formulation based on the principle of energy conservation [32]:


$$\rho_{f}AC_{p}\frac{\partial T}{\partial t}+\rho_{f}AC_{p}u\cdot \nabla T=\nabla \cdot Ak\nabla T+\frac{f_{D}\rho_{f}A}{\text{2}d_{h}}|u{|}^{\text{3}}+Q_{g}+Q_{Wall}$$ (9)

The radial heat transfer from the surrounding rock mass to the karst conduit is given by the following equation:


$$Q_{Wall}=(hZ{)}_{eff}(T_{ext}-T)\;$$ (10)

where the term *(hZ)*_*eff*_ represents the effective value of the heat transfer coefficient *h* (SI unit: W/m²·K) multiplied by the pipe wall perimeter *Z*; *T*_*ext*_ denotes the temperature of the surrounding rock mass external to the conduit.

#### 2.2.2 Heat conduction in surrounding rock.

In this study, the rock surrounding the karst conduit is considered as a homogeneous porous medium. By making the local thermal equilibrium hypothesis for solid and fluid phases, the Heat Transfer in Porous Media Interface solves for the following version of the heat equation [33], reformulated using a common temperature, T:


$${\left(\rho C_{p}\right)}_{eff}\frac{\partial T}{\partial t}+\rho_{f}C_{p,f}u\cdot \nabla T+\nabla \cdot q=Q\;$$ (11)

$$q=-k_{eff}\nabla T$$ (12)

where ρ_f_ is the fluid density; C_p,f_ is the fluid heat capacity at constant pressure; (ρC_p_)_eff_ is the effective volumetric heat capacity at constant pressure, defined by (ρC_p_)_eff_ = θ_s_ρ_s_C_p,s_ + ε_p_ρ_f_C_p,f_. Here, εp is the porosity; θ_s_ is the solid matrix volume fraction; ρ_s_ is the solid matrix density; C_p,s_ is the solid matrix heat capacity at constant pressure; k_eff_ is the effective thermal conductivity (a scalar or a tensor if the thermal conductivity is anisotropic); q is the conductive heat flux; **u** is the velocity field, either an analytic expression or computed from a fluid flow interface.

In the shallow subsurface rock layers (upper boundary, as shown in Fig 3), heat exchange occurs with the ground surface. The convective heat flux on the boundary in contact with the fluid (air or surface water) is then modeled as being proportional to the temperature difference across a fictitious thermal boundary layer. Mathematically, the heat flux is described by the equation:

**Fig 1:**
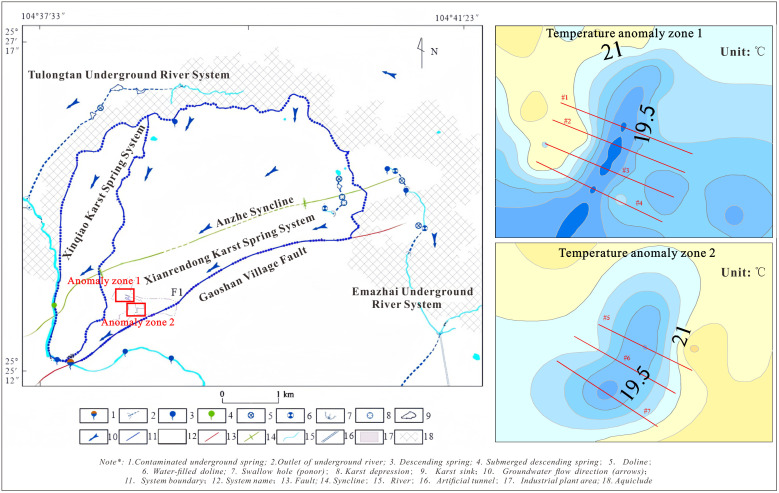
Overview of the study area. (1) Distribution of karst systems in the study area; (2) Temperature distribution in Geothermal Anomaly Zone 1; (3) Temperature distribution in Geothermal Anomaly Zone 2.

**Fig 2:**
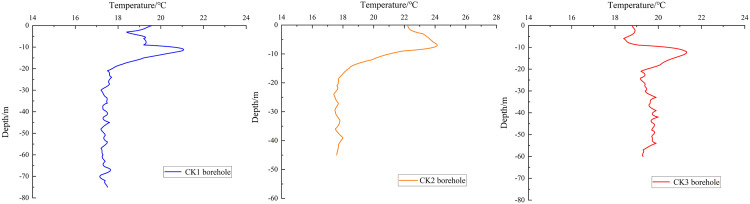
Temperature Variation with Depth in Investigation Boreholes CK1, CK2, and CK3.


$$-n\cdot q=h(T_{ext}-T)$$ (13)

where h is a heat transfer coefficient and Text the temperature of the external fluid far from the boundary.

### 2.3 Theory for Estimating Initial Parameters of Karst Groundwater Channels

For simplified water-bearing karst structure models, parameters such as the scale and burial depth of the structure can theoretically be inferred based on the shallow geothermal field. Tohara Kohzo investigated this issue and derived a simplified calculation formula for the shallow geothermal field [33]. Considering a horizontal cylindrical water-bearing karst structure in a semi-infinite homogeneous medium, it is assumed to be infinitely long with its central axis parallel to the Y-axis. By neglecting axial heat transfer, the problem is simplified to a two-dimensional plane. On the cross-section perpendicular to the cylinder axis, the X-axis is defined as parallel to the ground surface, and the Z-axis is vertically downward, passing through the center of the cylinder. If the burial depth of the cylinder center is h and the radius is r, the parameter *a* can be expressed as:


$$a=\sqrt{h^{\text{2}}-r^{\text{2}}}\;$$ (14)

The heat conduction equation and boundary conditions governing the steady-state temperature distribution in shallow subsurface formations, with surface convective heat exchange, are expressed as:


$$\{\begin{array}{c} {}*20c\frac{\partial^{2}T}{\partial x^{2}}+\frac{\partial^{2}T}{\partial z^{2}}=0\;\;\;\;\;\;\\ \frac{\partial T}{\partial z}{|}_{z=0}=\alpha T{|}_{z=0\;\;\;\;\;\;\;}\\ T{|}_{{\left(h-z\right)}^{2}+x^{2}=r^{2}}=T_{b} \end{array}$$ (15)

where *α* is the surface heat transfer coefficient, and T_b_ actually represents the temperature difference between the cylinder and ground surfaces.

When the ground surface can be considered as maintaining a constant temperature T_0_ (conventionally defined as zero for convenience), the boundary value problem governing the two-dimensional steady-state temperature field in shallow strata must satisfy the following conditions:


$$\{\begin{array}{c} {}*20c\frac{\partial^{2}T}{\partial x^{2}}+\frac{\partial^{2}T}{\partial z^{2}}=0\;\;\;\;\;\;\;\;\;\\ T{|}_{z=0}=T_{0}=0\;\;\;\;\;\;\\ T{|}_{{\left(h-z\right)}^{2}+x^{2}=r^{2}}=T_{b} \end{array}$$ (16)

Solving Equation (15) gives:


$$T=\frac{T_{b}}{ln\frac{h+a}{h-a}}\left[ln\frac{{r_{\text{2}}}^{\text{2}}}{{r_{\text{1}}}^{\text{2}}}+\text{4}\sum_{n=\text{1}}^{\infty }\frac{(-\text{1}{)}^{n-\text{1}}(n-\text{1})!\text{cos}\left(n\theta_{m}\right)}{\alpha^{n}r^{n}}\right]$$ (17)

where,


$$r_{\text{2}}=\sqrt{(z+a{)}^{\text{2}}+x^{\text{2}}}\;$$ (18)

$$r_{\text{1}}=\sqrt{(z-a{)}^{\text{2}}+x^{\text{2}}}$$ (19)

$$\theta_{m}={\text{tan}}^{-\text{1}}\left(\frac{x}{z+a}\right)\;$$ (20)

And solving Equation (16) gives:


$$T=\frac{T_{b}}{ln\frac{h+a}{h-a}}ln\frac{{r_{\text{2}}}^{\text{2}}}{{r_{\text{1}}}^{\text{2}}}$$ (21)

This study primarily targets the thermal regime at 2m depth. Substituting z = 2m into Eq. (17) and Eq. (21) yields the computational formulations for the 2m-depth temperature field under varying conditions:


$${T^{\prime }}_{z=\text{2}}=\frac{T_{b}}{ln\frac{h+a}{h-a}}\left[ln\frac{{\left(\text{1}+a\right)}^{\text{2}}+x^{\text{2}}}{{\left(\text{1}-a\right)}^{\text{2}}+x^{\text{2}}}+\frac{\text{4}(\text{1}+a)}{\alpha \left[(\text{1}+a{)}^{\text{2}}+x^{\text{2}}\right]}\right]$$ (22)

$${T^{\prime }}_{z=\text{2}}=\frac{T_{b}}{ln\frac{h+a}{h-a}}ln\frac{{\left(\text{1}+a\right)}^{\text{2}}+x^{\text{2}}}{{\left(\text{1}-a\right)}^{\text{2}}+x^{\text{2}}}\;$$ (23)

T'_z = 2_ denotes the thermal anomaly at 2m depth, defined as the difference between the temperature influenced by groundwater flow and the background subsurface temperature. Under the isothermal surface assumption, the inflection points *x*_*i*_ in the 2m-depth thermal curve are obtained as:


$$x_{i}=\frac{a}{\sqrt{\text{3}}}$$ (24)

The determination of *x*_*i*_ enables the solution for parameter a. By substituting *x* = *x*_*i*_ into Equation (22) and x* *= 0 into Equation (23), we obtain:


T′lx=xiz=2=T′i=Tblnh+ah−aln(1+a)2+xi2(1−a)2+xi2     (25)

Tlx=0z=2′=T0′=Tblnh+ah−aln(1+a)2(1−a)2 (26)

For the case where |a| is significantly greater than 2 m (|a| ≫ 2 m), we obtain:


$$\frac{T_{i}^{\prime }}{T_{\text{0}}^{\prime }}\approx \text{0}.\text{9}\text{6}\text{7}$$ (27)

The *T*_*x=2*_*-X* contour map of shallow subsurface temperature distribution, obtained from actual geothermal measurements in the study area, indicates that *T'*_*0*_ corresponds to the maximum anomaly point. Applying Equation (27) yields *T'*_*i*_, which enables the determination of *x*_*i*_. Subsequently, parameter *a* can be calculated using Equation (24), as follows:


$$\text{ln}\frac{h+a}{h-a}=\frac{T_{b}}{T_{\text{0}}^{\prime }}\text{ln}\frac{(\text{1}+a{)}^{\text{2}}}{(\text{1}-a{)}^{\text{2}}}$$ (28)

Under the convective boundary condition at the surface and for the case of |a|, |x| ≫ 2 m, Equation (22) reduces to:


$${T^{\prime }}_{z=\text{2}}=\frac{T_{b}}{\text{ln}\frac{h+a}{h-a}}\cdot \frac{\text{4}(\text{1}+a)}{\alpha \left[a^{\text{2}}+x^{\text{2}}\right]}$$ (29)

According to (d²T′/dx²) = 0, the inflection point coordinate can be determined as *x*_*i* _= a/√3, where the temperature ratio between this point and the origin *T*′*_i_*/ *T*′_*0*_ ≈ 0.967. Consequently, by substituting *x* = 0 into Eq. (22), we obtain:


$$\text{ln}\frac{h+a}{h-a}=\frac{T_{b}}{T_{\text{0}}^{\prime }}\left[\text{ln}\frac{(\text{1}+a{)}^{\text{2}}}{(\text{1}-a{)}^{\text{2}}}+\frac{\text{4}}{\alpha (\text{1}+a)}\right]$$ (30)

In conclusion, the parameters *x*_*i*_, *a*, *h*, and *r* can be fully determined given the convective heat transfer coefficient α and the temperature difference *T*_*b*_ between groundwater and the undisturbed ground surface. Here, *h* represents the burial depth of the water-bearing karst structure, while *r* denotes the effective flow radius of the karst conduit.

## 3. Study area

### 3.1 Field data

#### 3.1.1 Geological setting.

The study area is located in the Puanzhai area of Gaoshan Village, Dashan Town, Panzhou City, Guizhou Province, with geographical coordinates ranging from 104°35′59″E to 104°41′26″E in longitude and 25°24′10″N to 25°28′14″N in latitude, as shown in Fig 1. The study area is characterized by two primary folds trending NW-SE and one distinct fault. The stratigraphy of the study area consists of the Quaternary System (Q), Middle Triassic Guanling Formation (T₂g), and Lower-Middle Triassic Jialingjiang Formation (T₁ ₋ ₂_j_), with karst structures primarily developed in the Middle Triassic Guanling Formation (T₂g). The T₂g karst aquifer demonstrates high water abundance, with the formation generally under near-saturated conditions. The groundwater exhibits excellent physicochemical characteristics: colorless, odorless, tasteless, and transparent. Notably, the groundwater temperature remains remarkably stable with minimal influence from atmospheric variations, maintaining a constant range of 14–16°C throughout the year. The annual temperature fluctuation is limited to 2–3°C, classifying it as a typical cold-water system according to hydrogeological standards.

Fig 1 illustrates the conceptual hydrogeological framework of the study area. The groundwater system is primarily recharged by atmospheric precipitation, with localized surface water recharge occurring only in two subterranean river subsystems. Regionally, groundwater flow exhibits a multi-directional pattern: from northeast and north to southwest and south. The dominant flow mechanism occurs through fissure networks, while conduit flow is restricted to specific segments of the underground river systems. The entire groundwater discharge converges into the Xiaohuangni River, with most springs and karst channels exhibiting concentrated outflow patterns.

#### 3.1.2 Temperature measurement data.

For the ground temperature measurements in the study area, we employed two methods to obtain: (1) the horizontal distribution of temperature at a 2-meter depth (denoted as *T*_*i*_), and (2) the variation of ground temperature with depth (denoted as *T*_*d*_). The 2-meter temperature *T*_*i*_ measurements were conducted using a self-developed multi-parameter thermometer capable of measuring at 2-meter depth, while the temperature *T*_*d*_ variation with depth was monitored using Distributed Optical Fiber Temperature Sensing (DTS) technology. The 2-meter survey points were mainly deployed in the NW and SE parts of the study area, where shallow karst formations are prominent. DTS measurements were conducted in boreholes CK1, CK2, and CK3 (Fig 2).

During long-term field investigations, measured values are influenced by annual variations in surface temperature, which exhibit a relatively complex pattern. To address this, fixed monitoring points were established with temperature recorded three times per day. Preliminary analysis of the temperature data revealed a difference of 0.23 °C between the first and second days. Based on this finding, corrections were applied to ensure consistent surface temperature across the monitoring points, and the correction results are presented in Table 1.

**Table 1:** Temperature Correction for Diurnal Variation.

Measurement Date	Timepoint	Weather Conditions	Measured Value (°C)	Mean Value (°C)	Corrected Value (Δ=±X, °C)
2023.10.25	8:32	Sunny	24.05	24.17	−0.07
	12:34		24.32		
	16:42		24.14		
2023.10.26	9:03	Sunny	23.82	23.94	+0.16
	13:24		24.05		

In addition, topographic factors such as slope aspect and elevation difference exert certain influences on shallow ground temperature. In the study area located in Panzhou City, Guizhou Province, the site has been leveled and is predominantly composed of north-west sloping and flat terrain. As a result, the effect of slope aspect on ground temperature can be considered negligible. The elevation difference within the area is approximately 3 m, which has a minor impact on the temperature at a depth of 2 m; therefore, elevation-related corrections were also omitted. However, geomorphological features significantly affect the ground temperature at 2 m depth. The temperature variation among different geomorphic units in the study area ranges from −1.66 °C to +1.57 °C. Considering the controlling effect of karst structures on temperature distribution, this variation is notable and requires appropriate adjustment. The surface types in the investigated area mainly include paved areas (B), rock areas (R), grassland (G), and areas under tree canopy (T). The corrected temperature data are summarized in Table 2.

**Table 2:** Surface feature-based temperature calibration.

Geomorphic feature	*T*_*max*_ (°C)	*T*_*min*_ (°C)	Mean Value (°C)	Corrected Value (Δ=±X, °C)	Proportion (%)
B	24.11	20.54	23.02	−1.66	10.24
R	23.16	18.67	22.28	−0.92	12.99
G	21.93	19.16	20.33	+1.03	57.34
T	21.45	18.28	19.79	+1.57	19.43
Total	/	/	/	/	100

Following the implementation of date-specific thermal corrections and geomorphic adjustment factors, we derived interference-free shallow subsurface temperature data. The corrected 2m-depth temperature results are presented in Table 3.

**Table 3:** Corrected results of shallow layer temperature measurement.

Measuring point	Longitude	Latitude	Temperature at 2-m Depth	Thermal Conductivity	Specific Heat
O	104.6176	25.41462	18.36	0.860356	693.667
O-5	104.6171	25.41482	20.09	0.493962	547.431
O-3	104.6174	25.41472	21.55	0.271122	884.831
O-4	104.6172	25.41477	20.09	0.19454	670.875
O-2	104.6175	25.41468	20.8	0.728559	867.093
N1-1	104.6176	25.4147	18.24	0.406268	988.772
N1-2	104.6175	25.41475	20.75	0.464324	679.076
N1-3	104.6174	25.41479	20.92	0.058413	917.53
N1-4	104.6173	25.41484	20.68	0.194383	970.386
N1-5	104.6172	25.41488	20.18	0.205791	964.781
N2-1	104.6176	25.41478	18.09	0.525723	641.529
N2-2	104.6175	25.41482	21.45	1.022261	643.729
N2-3	104.6174	25.41487	21.33	0.085086	842.134
N2-4	104.6173	25.41491	21.73	0.278695	880.346
N2-5	104.6172	25.41495	20.39	0.518975	671.437
N3	104.6177	25.41487	18.36	0.278695	880.362
N4	104.6177	25.41498	18.71	0.402675	911.212
N5	104.6178	25.41508	18.98	0.264131	743.213
N7	104.6178	25.41523	21.05	0.458791	690.256
N6	104.6178	25.41517	21.16	0.335743	700.014
N9	104.6176	25.4153	20.67	0.295284	822.122
N8	104.6176	25.41523	21.65	0.277768	560.132
N10	104.6175	25.41528	20.23	0.393449	880.231
N11	104.6175	25.41535	20.79	0.357095	976.272
N12	104.6174	25.41538	21.86	0.30248	811.231
E1-1	104.6177	25.41459	20.25	0.871218	950.125
E1-4	104.6181	25.41442	20.11	0.402983	862.345
E1-2	104.6178	25.41453	19.64	0.265853	745.623
E1-3	104.6179	25.41447	19.68	0.704809	962.312
E2-1	104.6177	25.41466	19.64	0.699715	845.326
E2-2	104.6178	25.41462	18.94	0.2529	768.524
E2-3	104.618	25.41456	19.73	0.835992	812.742
E2-4	104.6181	25.41451	18.67	1.244648	697.824
E-3	104.6177	25.41475	19.64	0.519599	842.369
E4	104.6178	25.41484	19.79	0.634434	965.251
E5	104.6178	25.41494	20.47	0.546429	959.671
E6	104.6179	25.41513	20.57	0.568429	765.312
E7	104.6182	25.41502	20.49	0.463821	959.671
S1-1	104.6171	25.41473	19.26	0.429893	865.365
S3	104.6173	25.41463	19.51	1.257871	624.612
S5	104.6175	25.41454	18.35	0.278325	942.165
S6	104.6177	25.41445	19.35	0.383349	984.134
S7	104.618	25.41432	19.28	0.718777	679.531
S8	104.6183	25.41422	20.16	0.063672	870.358
S9	104.6186	25.41407	20.33	0.581833	924.421
S10	104.6189	25.41385	20.71	0.415443	941.652
S11	104.619	25.4136	21.83	0.429514	660.125
S12	104.619	25.41335	21.96	0.555856	690.185
S13	104.6191	25.41311	21.19	0.379446	763.523
S14	104.6191	25.41288	20.64	0.48936	863.541
S15	104.6192	25.4127	18.54	0.757458	860.162
S17	104.6192	25.41216	24.11	0.457456	741.267
S18	104.619	25.41207	22.28	0.835946	632.642
K1	104.6197	25.41436	22.79	0.303668	765.364
K2	104.6199	25.41452	22.07	0.873723	842.135
S16	104.6192	25.41251	21.28	1.151764	682.31
S2	104.6172	25.41468	19.57	0.725665	570.039
S4	104.6174	25.41459	18.99	1.059447	551.265
H1-1	104.6193	25.41301	19.14	0.496989	862.124
H1-2	104.6195	25.41301	21.23	0.61248	675.312
H2-1	104.6193	25.41288	18.93	0.47265	812.685
H2-2	104.6195	25.41288	22.11	0.699016	763.189
H2-3	104.6197	25.41286	21.08	0.869338	821.231
H4.5	104.6201	25.41285	21.3	0.954539	689.371
H4	104.6199	25.41286	22.02	0.936751	902.372
H5	104.6203	25.41283	20.59	1.173914	880.316
H6	104.6206	25.41283	20.96	1.304422	791.379
N13	104.6173	25.41534	22.65	0.24368	632.872
N14	104.6173	25.41544	21.23	0.753906	800.359
N15	104.6172	25.41539	21.89	0.902493	950.345
N16	104.6171	25.41547	19.59	0.265797	590.189

Fig 2 presents the temperature variation curves versus depth, as measured by DTS technology in monitoring boreholes CK1, CK2, and CK3. Affected by temperature variations, significant geothermal fluctuations were observed within the 0–10 m depth range in all three boreholes, below which a clear decreasing trend in ground temperature was observed. According to research by Chi et al. [33], this type of geothermal distribution pattern can be classified as a “gradual cooling-type” formation. The formation mechanism of this phenomenon may be related to shallow groundwater activity: when groundwater at lower temperatures undergoes horizontal flow and vertical infiltration, it significantly reduces the geothermal gradient in the affected strata. Borehole temperature profiles reveal distinct minimum temperature depths at ~27 m (CK3) and 20 m (CK1 and CK2), indicating a probable horizontal groundwater flow zone centered within this depth interval. To better constrain the reliability of karst structure characterization through geothermal anomaly analysis, numerical modeling will be implemented in subsequent studies.

## 4. Numerical modeling

### 4.1 Geometrical model

To investigate the feasibility of applying shallow geothermal prospecting to karst structure surveys, this study developed a numerical model of shallow formation temperature distribution in water-bearing karst systems. The model was used to analyze various factors affecting shallow geothermal distribution patterns and to estimate the effective detection depth of karst structures based on shallow geothermal anomalies.

**Fig 3:**
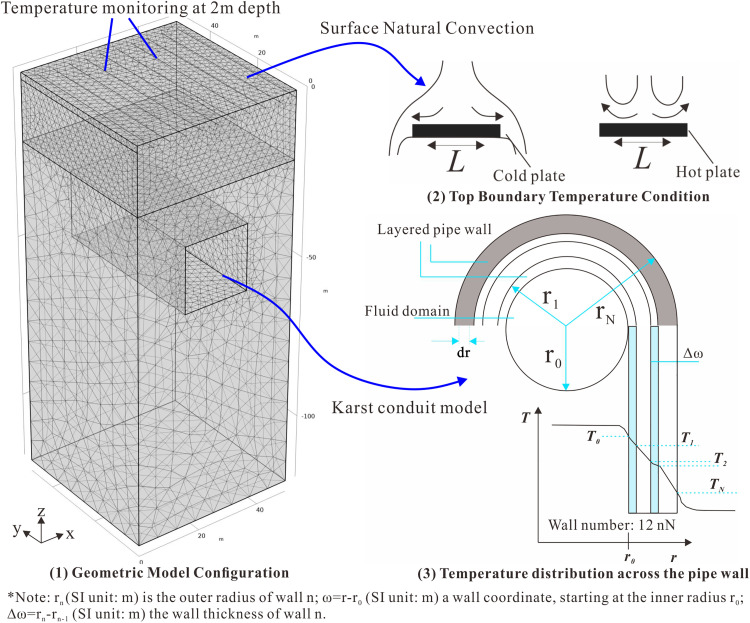
Geometric model and boundary condition setup for karst structures: (1) geometric model; (2) top boundary temperature condition; (3) temperature distribution across the pipe wall [34].

Fig 3 illustrates the geometric configuration and mesh discretization of the numerical model. The domain consists of a three-dimensional structure measuring 60 m (x) × 60 m (y) × 100 m (z). A localized 10 m × 10 m square region is incorporated within the yz-plane to simulate the spatial characteristics of the karst development zone. Since the study area strata primarily consist of Guanling Formation limestone, the model simplifies the entire Guanling Formation as a homogeneous stratum with uniform thermophysical properties, while disregarding the influence of residual soil cover. To accommodate the requirements of different numerical models, the mesh generation employs a physics-controlled adaptive triangular auto-partitioning method, with particular refinement around karst channels using mapped meshing technique to enhance model accuracy. Additionally, grid densification was implemented within the 20-meter-deep thermocline layer to more precisely simulate temperature distribution in this zone. The model parameters were determined based on laboratory measurements and in-situ testing data, as summarized in Table 4.

**Table 4:** Modeling parameters determined from laboratory measurements and field test data.

Parameters	Value
Pipe fluid thermal conductivity/W·m ⁻ ¹·K ⁻ ¹	0.6
Model top initial temperature/°C	15.2
Rock porosity/%	1.0
Rock permeability/m²	1 × 10^-15^
Fracture seepage velocity/ (m·s ⁻ ¹)	0.269
Rock thermal conductivity/ (W·m ⁻ ¹·K ⁻ ¹)	2.0
Rock specific heat/ (J·kg ⁻ ¹·K ⁻ ¹)	800

### 4.2 Initial and boundary conditions

The upper boundary was assigned a constant hydraulic head (H = 120 m) to represent the sustained recharge from the Xiaohuangni River. This configuration, combined with uniform vertical infiltration, enables the model to capture both the steady-state groundwater flow regime and its thermohydrogeological coupling effects in the saturated karst formation.

The model assumes steady heat conduction in the rock mass with convective cooling at the surface. Initial conditions were derived from field measurements, with geothermal gradients approximated by the lower boundary temperature (18.38°C). The identical rock and air temperatures (15.2°C) at t = 0 represent equilibrium conditions before thermal perturbation. The upper boundary represents the rock outcrop at the ground surface, where convective heat transfer occurs with the air, while thermal radiation and other heat transfer mechanisms are neglected, as shown in Fig 3 (2). These simplified boundary conditions allow the simulation to focus primarily on internal heat conduction and convection processes within the rock mass, thereby improving computational efficiency. The convective heat flux at this boundary satisfies Equation (31):


$$-\lambda {\left(\frac{\partial T}{\partial n}\right)}_{W}=K_{a}\left(T_{W}-T_{f}\right)$$ (31)

where *λ* is the thermal conductivity of the rock matrix, *Ka* denotes the thermal conductivity of air, and *n* represents the normal distance to the isothermal surface.

The groundwater flow in the equivalent karst conduit system was simulated using constant-pressure and constant-velocity boundary conditions, coupled with a wall heat transfer model to characterize thermal exchange between the conduit and surrounding rock matrix, as shown in Fig 3 (3). The heat transfer mechanism between the karst conduit and surrounding rock matrix is rigorously described by Equation (9).

### 4.3 Analysis of numerical simulation results

#### 4.3.1 Impact of karst structure burial depth.

**Fig 4:**
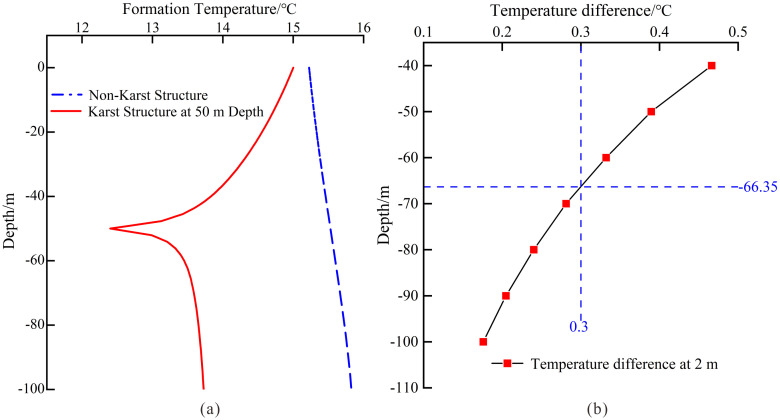
Thermal Profiles and Differences Between Karst and Non-Karst Structures. (a) Comparison of temperature profiles between a karst structure at 50 m depth and and non-karst formation; (b) temperature difference curve between karstic and non-karstic bodies at 2 m depth.

Given the relatively homogeneous stratigraphy within the study area’s depth range – predominantly composed of the Guanling Formation’s limestone without significant interbedding, so we established a homogeneous limestone model as the baseline case for comparative analysis. The homogeneous limestone model reveals fundamental thermal stratification patterns unaffected by karst features. The quasi-linear profile below 20 m depth (Fig 4a) establishes the reference geothermal gradient, against which karst-induced perturbations can be quantified.

When karst structures containing low-temperature water (12°C) are present in the formation, they fundamentally alter the thermal distribution pattern, creating distinct inflection points in the temperature-depth profile. Fig 4a illustrates this phenomenon through the geothermal gradient observed in a 50 m-deep karst conduit system. Fig 4a shows the geothermal temperature distribution curve under the condition of a karst structure at a burial depth of 50 m. As can be seen from Fig 4a, a karst structure containing low-temperature fluid alters the formation temperature distribution. Under the influence of the karst structure, the formation temperature first decreases, with the temperature gradient increasing as it approaches the structure. The temperature reaches its lowest point at the karst structure and then rapidly recovers between 50–60 m. Beyond a burial depth of 70 m, the temperature gradient stabilizes and becomes consistent with that of formations without a karst structure (Baseline case). These findings confirm that karst structures exert a measurable influence on subsurface thermal regimes.

To systematically investigate the influence of karst structure burial depth on the shallow temperature field at 2m depth, we varied the burial depth of karst channels within a range of 40–100 meters in our numerical model. As illustrated in Fig 4b, the temperature differential at 2m depth between formations with and without karst structures increases nonlinearly as the burial depth of the karst structure decreases. This phenomenon demonstrates that shallower karst structures exert more pronounced thermal impacts on near-surface formations. The study employed shallow geothermal measurement equipment with an inherent measurement error margin. Based on instrument specifications, temperature variations exceeding 0.3°C were considered reliably detectable, thus establishing 0.3°C as the observation threshold. Under experimental conditions featuring a single karst structure with 0.5 m equivalent diameter and 12°C water temperature, the effective detection range of the equipment was determined to be within 66m depth. Beyond this critical depth, the thermal anomalies induced by karst structures diminish below the detection threshold, resulting in ambiguous identification results.

#### 4.3.2 Impact of karst conduit equivalent diameter.

The study area exhibits a zone (0–100 m depth) characterized by an intermingling of shallow phreatic caves and deep phreatic caves. The deep phreatic channels typically display elliptical cross-sections measuring 1–2 m, while the shallow phreatic channels possess comparatively smaller cross-sectional dimensions. To account for variations in effective flow cross-sectional areas of karst structures, the modeling setup standardized the burial depth at 50 m and fluid temperature at 12°C. Using the dry-season discharge as the minimum flow condition, numerical models were constructed with equivalent diameters (*d*) ranging from 0.1 to 1.0 m. Under constant flow conditions (q = 52.86 L/s), the corresponding flow velocities for each equivalent diameter are presented in Table 5.

**Table 5:** Flow velocity versus equivalent diameter.

Equivalent diameter/m	0.1	0.2	0.3	0.4	0.5	0.6	0.7	0.8	0.9	1.0
Flow velocity/m·s ⁻ ¹	6.730	1.683	0.748	0.421	0.269	0.187	0.137	0.105	0.083	0.067

**Fig 5:**
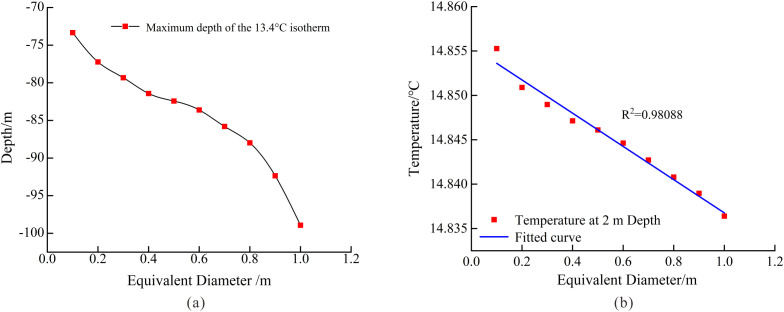
Inferring Karst Conduit Scale from Shallow Thermal Signals. (a) Relationship between the maximum depth and equivalent diameter of the 13.45°C isotherm; (b) Variation of subsurface temperature at 2 m depth with karst equivalent diameter.

Fig 5a reveals the quantitative relationship between the equivalent diameter of karst structures and the depth of geothermal temperature influence. Numerical simulation results clearly demonstrate that as the equivalent diameter increases, the disturbance range of the karst structure on the formation temperature field exhibits significant expansion. Specific data indicate that when the equivalent diameter increases from 0.1 m to 0.5 m, the maximum depth of temperature influence rises from an initial 73 m to 83 m, representing a 13.7% increase. Further increasing the diameter to 1 m results in a maximum influence depth of 98 m, corresponding to an overall 34.2% enhancement compared to the 0.1 m baseline. This nonlinear growth pattern suggests that the enlargement of karst dimensions not only linearly extends their thermal influence range but also substantially amplifies thermal disturbance in surrounding formations through enhanced thermal convection effects.

Fig 5b presents a comparative analysis of formation temperature distribution characteristics at 2m depth under constant flow rate (q = 52.86 L/s) but varying equivalent diameters (d = 0.1-1.0m). The results reveal three significant findings:1) Under constant flow conditions, equivalent diameter variation exhibits limited influence on shallow geothermal temperature: a tenfold diameter increase (0.1m → 1.0m) results in merely 0.02°C temperature rise; 2) Velocity variation (v = 0.64–6.37 m/s) shows no discernible temperature gradient effect; 3) Temperature fluctuations (ΔT < 0.02°C) remain below the identifiable observation threshold. We attribute these phenomena to the strong thermal diffusivity of shallow formations, which effectively buffers thermal disturbances induced by fluid movement.

**Fig 6:**
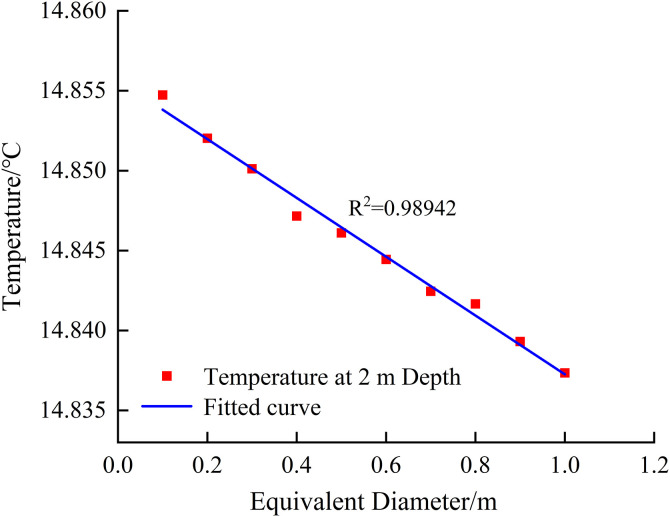
Temperature at 2 m depth as a function of equivalent karst diameter for a flow velocity of 0.269 m/s.

To further investigate the influence of groundwater flow in karst channels on formation temperature, this study adopted simulation conditions with a fixed flow velocity (u = 0.269 m/s), which implies different discharge values corresponding to karst channels of varying equivalent diameters. Fig 6 illustrates the characteristics of temperature influence at 2 m depth under constant flow velocity conditions with changing equivalent diameters. Comparative analysis with the variable-velocity results in Fig 5b reveals: (1) Under identical discharge conditions, the temperature influence difference caused by varying flow velocities in karst structures does not exceed 0.002°C; (2) Similarly, under constant flow velocity conditions, variations in equivalent diameter (d = 0.1–1 m) exhibit negligible effects on shallow geothermal temperature. These results demonstrate that the influence of groundwater flow parameter variations on shallow temperature measurements is insignificant. Consequently, when the equivalent diameter of karst channels ranges between 0.1–1 m, shallow temperature measurement methods prove ineffective in identifying variations in flow cross-sectional characteristics.

#### 4.3.3 Impact of karst groundwater temperature.

Building upon the established numerical model of the karst structure (equivalent diameter d = 0.5 m, burial depth = 50 m), we investigated groundwater temperature variations from 12 to 18°C (characteristic temperature range for karst groundwater). Employing a 0.5°C temperature increment, we systematically examined the characteristic influences of fluid temperature variations on the formation’s thermal field.

**Fig 7:**
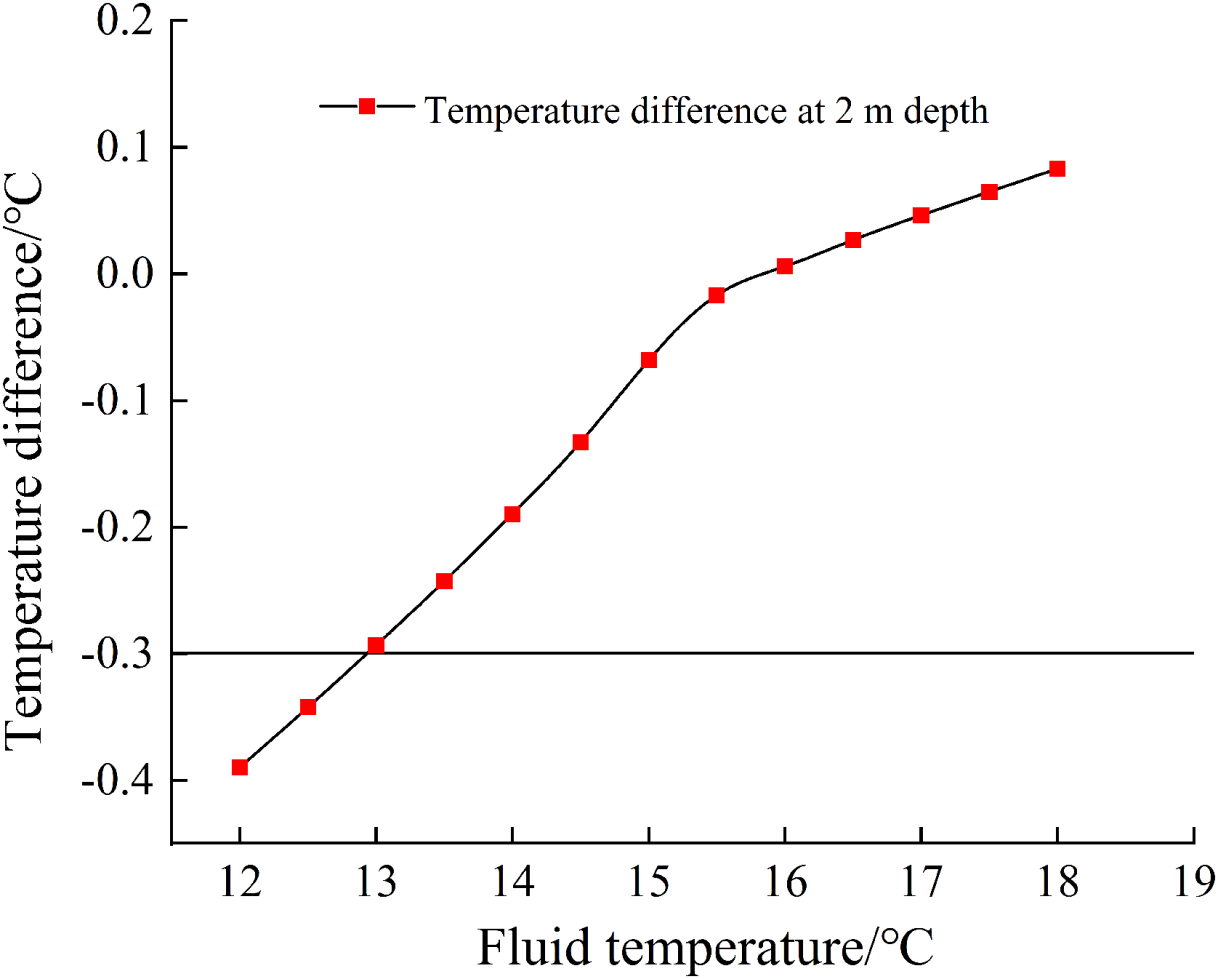
Temperature difference curve at 2m depth: karst vs. non-karst under various fluid temperatures.

Fig 7 compares the temperature differences at 2 m depth between scenarios with and without the karst structure under varying fluid temperature conditions. The results demonstrate that the thermal contrast between groundwater temperature in the karst structure and the original rock temperature significantly affects shallow geothermal field distribution:1) When fluid temperature is lower than the original formation temperature (15.5 °C), the shallow formation exhibits a cooling effect, showing a linear temperature difference curve with a slope of 0.107; 2) When fluid temperature exceeds the formation temperature, the shallow formation demonstrates a heating effect, with the curve slope decreasing to 0.038; 3) A distinct inflection point occurs near 15.5 °C (initial formation temperature), indicating this value represents the critical threshold for thermal influence. These findings reveal that low-temperature fluids produce significantly greater perturbations in shallow geothermal fields than high-temperature fluids. Using 0.3 °C as the detection threshold for temperature anomalies, the shallow geothermal measurement method can effectively identify karst structures (0.5 m diameter at 50 m depth) when fluid temperatures fall below 13 °C.

## 5 Thermal signature analysis for karst feature identification

Numerical simulations reveal that, under the hydrogeological conditions of the study area, the influence of flow velocity variations within karst channels on shallow subsurface temperature distribution is negligible. Guided by this finding, we simplified the conduit flow representation in the original three-dimensional (3D) parameter estimation model, while preserving both the model’s validity and the established boundary conditions. This dimensionality reduction significantly improves computational efficiency without sacrificing analytical rigor. Subsequently, we apply parameter estimation constrained by shallow geothermal measurement data to identify the burial depth and spatial characteristics of karst structures. The BOBYQA (Bound Optimization BY Quadratic Approximation) algorithm is employed for this purpose, with convergence criteria set to a temperature change (ΔT) of less than 0.01°C and parameter variations below 2% per iteration.

To account for the observed vertical heterogeneity in thermophysical properties despite lithological homogeneity in the study area, we developed a stratified 3D parameter estimation model comprising four distinct layers to enhance computational accuracy. Maintaining consistency with our 3D numerical simulations, we employed the same physics-controlled adaptive triangular meshing scheme, which automatically optimizes grid density based on thermal gradient variations. The initial parameters for the estimation model, including the burial depth and effective flow radius of karst structures, were determined using the theoretical framework established by Tohara Kohzo, with the resultant values presented in Table 6.

**Table 6:** Estimated initial parameter values of karst structures for each survey line.

Line ID	1	2	3	4	5	6	7
Central depth/m	35.25	38.67	39.64	41.13	44.53	46.27	43.61
Effective flow radius/m	3.94	3.88	3.92	4.06	4.84	4.92	4.73

**Fig 8:**
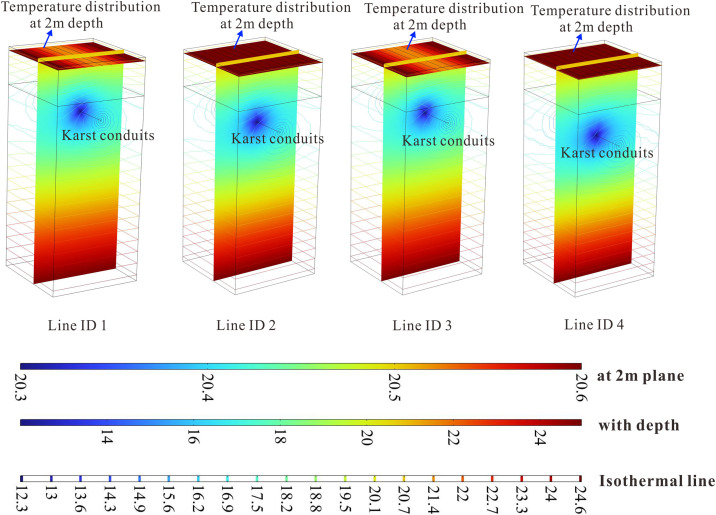
Spatial distribution of estimated geothermal temperature along various survey lines in Low-Temperature Anomaly Zone 1 derived by parameter estimation.

**Fig 9:**
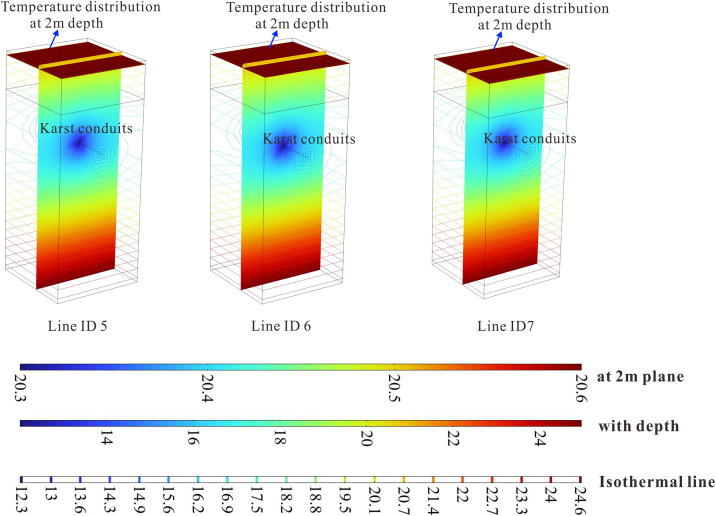
The spatial distribution of geothermal isotherms derived from parameter estimation across all survey lines in Low-Temperature Anomaly Zone 2.

Figs 8 and 9 presents the spatial distribution of geothermal isotherms derived from parameter estimation across all survey lines. It can be clearly observed that the presence of low-temperature runoff in karst channels causes significant upward protrusions in the isothermal contours, resulting in pronounced local thermal anomalies at shallow depths (typically within 2–5 meters). This phenomenon manifests as oval-shaped isotherms surrounding the karst channels that become sharply distorted at their upper sections. Such hydrothermal signatures serve as diagnostic evidence of active karst groundwater systems, where descending cold water flows through permeable channels create persistent heat-absorption zones. The spatial configuration of these thermal anomalies shows strong correlations with: (i) the burial depth of karst structures, and (ii) the equivalent flow radius.

**Fig 10:**
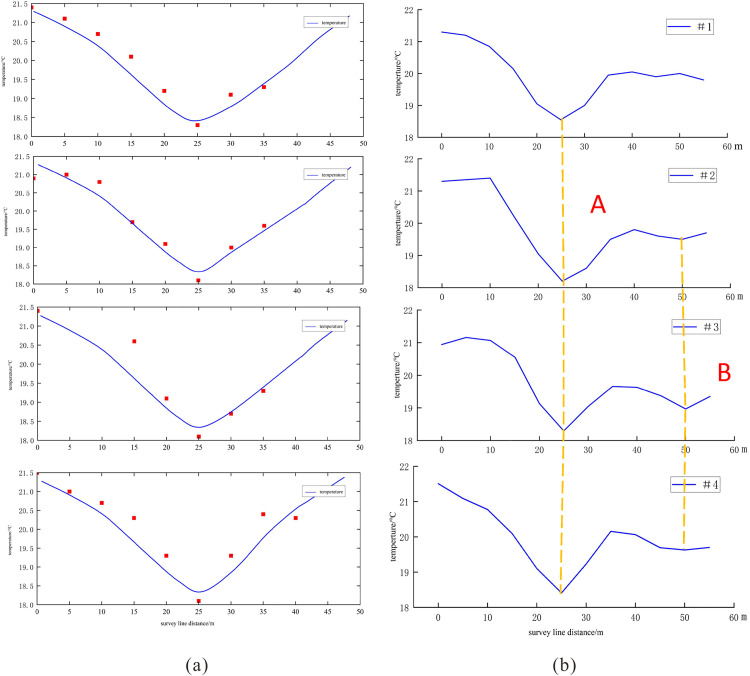
Temperature Profile Fitting and Inferred Karst Conduits in Anomaly Zone 1. (a) the fitting results of four 2m-depth temperature profiles in Low-Temperature Anomaly Zone 1; (b) potential karst channel locations inferred based on temperature profiles.

Fig 10 presents the fitting results of four 2m-depth temperature profiles in Low-Temperature Anomaly Zone 1. A comparison with the measured temperature curves reveals that the model, which assumes a homogeneous limestone formation, provides a good fit for Main Conduit A but fails to accurately reproduce the thermal signature of Secondary Conduit B. This discrepancy arises because small-scale karst features (e.g., stalagmites and stone teeth) near Conduit A in the actual geological setting have developed into minor low-temperature zones (Conduit B), locally perturbing the shallow thermal distribution across Anomaly Zone 1. As a result, the measured shallow geothermal profiles (Survey Lines 1–4) exhibit asymmetric distributions with distinct minima rather than the expected symmetrical pattern. Consequently, a homogeneous geological model cannot simultaneously predict the thermal effects of dual karst channels with high accuracy.

**Fig 11:**
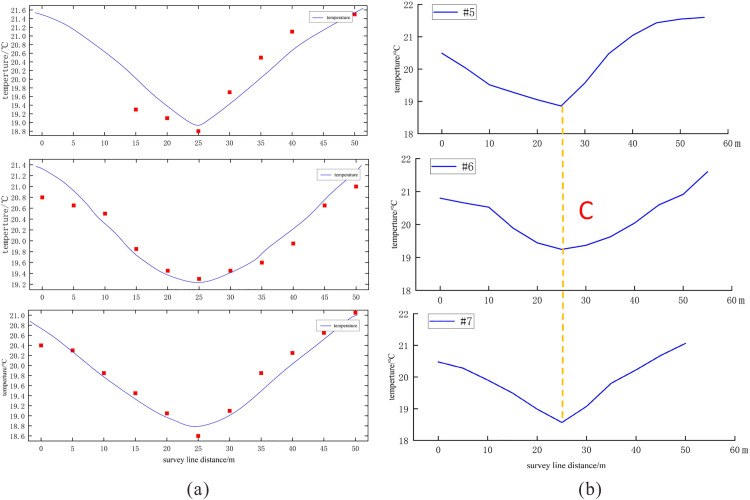
Temperature Profile Fitting and Inferred Karst Conduits in Anomaly Zone 2. (a) The fitting results of three 2m-depth temperature profiles in Low-Temperature Anomaly Zone 2; (b) potential karst channel locations inferred based on temperature profiles.

Fig 11 displays the simulated and measured temperature profiles along three survey lines at a 2 m depth in Low-Temperature Anomaly Zone 2. The model demonstrates strong agreement with the field measurements, indicating its capability to reliably estimate key karst parameters, including the burial depth and spatial scale of the underlying karst structure. The strong agreement between the simulated and observed thermal profiles indicates that the thermal anomaly pattern is predominantly governed by a well-structured karst conduit system, rather than dispersed small-scale heterogeneities. This robust model validation further demonstrates that low-temperature anomalies in karst systems can serve as reliable indicators for reconstructing subsurface conduit networks, particularly in geological settings amenable to simplified modeling approaches.

Through parameter estimation using BOBYQA, with convergence criteria set as ΔT < 0.01°C and parameter variations < 2% per iteration, we successfully determined the characteristic parameters of karst structures (burial depth of karst channels and effective flow radius) under various survey line conditions, as shown in Table 7. Specifically, in Anomaly Zone 1, the main karst conduit had a burial depth of 32.65–37.27 m and an effective flow radius of 3.81–4.04 m. In Anomaly Zone 2, the karst conduit exhibited a burial depth of 37.46–40.15 m and an effective flow radius of 4.09–4.91 m. The estimated results are in good agreement with the observations from borehole exploration and temperature logging (karst conduit burial depth: 30–40 m). This confirms the feasibility of inverting the burial depth and scale of underground karst channels using shallow (2 m depth) temperature measurements, providing a novel approach for the investigation of deep-seated geological hazards.

**Table 7:** Burial depth and flow radius of karst structures from different survey lines.

Line ID	1	2	3	4	5	6	7
Central depth/m	33.6	32.65	37.27	36.33	39.05	40.15	37.46
Effective flow radius/m	3.95	3.89	3.81	4.04	4.09	4.91	4.48

## 6 Conclusions

This paper conducts an in-depth study on the numerical simulation and parameter estimation of shallow geothermal measurements for investigating water-bearing karst structures, taking the Panzhou study area as an example. Relevant field test data were collected to verify the feasibility and effectiveness of the method. The main conclusions are as follows:

1) This study simulates shallow geothermal fields influenced by water-bearing karst structures to assess their detectability via shallow temperature surveys. Results show that shallow strata temperature non-linearly decreases with shallower karst structure burial depth. While the effective flow area significantly impacts the temperature distribution, variations in individual karst conduits’ equivalent diameters cause negligible temperature changes (<0.02°C at 2m depth), making them undetectable. However, treating multiple structures as an integrated equivalent cross-section enhances detection efficacy. Low-temperature fluids have a more pronounced cooling effect, and a 0.3°C threshold allows detection of a 50m deep, 0.5m diameter structure when its fluid temperature is below 13°C.
2) Based on 2-meter-deep stratum temperature data, a parameter estimation model was developed to characterize water-bearing karst structures. First, by integrating theoretical methods for estimating initial karst parameters, a shallow stratigraphic thermal model incorporating karst features was established. The geothermal profile at 2 m depth was simulated using finite element analysis. The measured temperature curve was then compared with the theoretical curve, and model parameters were iteratively refined to achieve an optimal fit. Finally, parameter optimization was performed using the BOBYQA (Bound Optimization BY Quadratic Approximation) algorithm, enabling accurate identification of the characteristics of shallow water-bearing karst structures.
3) Based on experimental data collected from the study area in Panzhou City, Guizhou Province, a parameter estimation model was applied to evaluate the development and burial depth of shallow water-bearing karst structures. The results indicate two main flow paths within the karst system, situated in Anomaly Zone 1 and Anomaly Zone 2, which extend from the northeast to the southwestern discharge area near the Xiaohuangni River. The effective flow radius ranges from 3.81 m to 4.91 m, and the depth of the karst structures varies between 32.15 m and 40.15 m. These findings are consistent with geophysical exploration results and distributed temperature sensing (DTS) measurements, further confirming the reliability and effectiveness of the parameter estimation method.

## Supporting information

S1 Fig 2 Temperature variation with depth in investigation boreholes CK1, CK2, and CK3. (ZIP)
